# Floral micromorphology and nectar composition of the early evolutionary lineage *Utricularia* (subgenus *Polypompholyx*, Lentibulariaceae)

**DOI:** 10.1007/s00709-019-01401-2

**Published:** 2019-06-12

**Authors:** Bartosz J. Płachno, Małgorzata Stpiczyńska, Piotr Świątek, Hans Lambers, Gregory R. Cawthray, Francis J. Nge, Saura R. Silva, Vitor F. O. Miranda

**Affiliations:** 1 Department of Plant Cytology and Embryology, Institute of Botany, Faculty of Biology, Jagiellonian University in Kraków, 9 Gronostajowa St., 30-387 Cracow, Poland; 2 Botanic Garden, Faculty of Biology, University of Warsaw, Al. Ujazdowskie 4, 00-478 Warsaw, Poland; 3 Department of Animal Histology and Embryology, University of Silesia in Katowice, 9 Bankowa St., 40-007 Katowice, Poland; 4 School of Biological Sciences, University of Western Australia, (M084) 35 Stirling Highway, Crawley Perth, WA 6009 Australia; 5 School of Biological Sciences, Faculty of Science, The University of Adelaide, Adelaide, South Australia 5005 Australia; 6 Faculdade de Ciências Agrárias e Veterinárias, Jaboticabal, Departamento de Biologia Aplicada à Agropecuária, Universidade Estadual Paulista (Unesp), São Paulo, Brazil

**Keywords:** Australian bladderwort, Bee pollination, Carnivorous plant, Floral micromorphology, HPLC, Lentibulariaceae, Nectary structure, Nectar composition, *Polypompholyx*, *Pleiochasia*, Spur, Trichomes

## Abstract

*Utricularia* (Lentibulariaceae) is a genus comprising around 240 species of herbaceous, carnivorous plants. *Utricularia* is usually viewed as an insect-pollinated genus, with the exception of a few bird-pollinated species. The bladderworts *Utricularia multifida* and *U. tenella* are interesting species because they represent an early evolutionary *Utricularia* branch and have some unusual morphological characters in their traps and calyx. Thus, our aims were to (i) determine whether the nectar sugar concentrations and composition in *U. multifida* and *U. tenella* are similar to those of other *Utricularia* species from the subgenera *Polypompholyx* and *Utricularia*, (ii) compare the nectary structure of *U. multifida* and *U. tenella* with those of other *Utricularia* species, and (iii) determine whether *U. multifida* and *U. tenella* use some of their floral trichomes as an alternative food reward for pollinators. We used light microscopy, histochemistry, and scanning and transmission electron microscopy to address those aims. The concentration and composition of nectar sugars were analysed using high-performance liquid chromatography. In all of the examined species, the floral nectary consisted of a spur bearing glandular trichomes. The spur produced and stored the nectar. We detected hexose-dominated (fructose + glucose) nectar in *U. multifida* and *U. tenella* as well as in *U. violacea*. In both *U. multifida* and *U. tenella*, there were trichomes that blocked the entrance into the throat and spur. Because these trichomes were rich in chromoplasts and contained lipid droplets, they may form an additional visual attractant. Bearing in mind the phylogenetic hypothesis for the genus, we suggest that an early ancestor of *Utricularia* had a nectariferous spur flower with a lower lip that formed a wide landing platform for bee pollinators.

## Introduction

The Lentibulariaceae comprise three monophyletic genera of carnivorous plants: *Pinguicula* L., *Genlisea* A.St.-Hil., and *Utricularia* L. (Juniper et al. 1989; Jobson et al. 2003). According to Silva et al. (2018), the last common ancestor of the *Genlisea*-*Utricularia* clade was a South American lineage that arose 39 million years ago (Mya). The genus *Utricularia* probably diverged from its sister genus *Genlisea* about 30 Mya and dispersed to Australia with the lineage that is represented by the subgenus *Polypompholyx*, about 17 Mya. According to Jobson et al. (2017), during the evolution of the subgenus *Polypompholyx* lineage, the first shift occurred c. 15 Mya during the mid-Miocene with the establishment of the two major lineages, one of them being the lineage that now represents the section *Polypompholyx* (*Utricularia multifida*, *U. tenella*) and the section *Tridentaria* (*Utricularia westonii*). In the past, *Polypompholyx* Lehm. [along with the species *Polypompholyx multifida* (R.Br.) F.Muell. and *Polypompholyx tenella* (R.Br.) Lehm.] was treated as a genus that was separate from the genus *Utricularia*; however, Taylor (1986) ranked *Polypompholyx* as a subgenus of *Utricularia*. The subgenus *Polypompholyx* includes four sections: *Polypompholyx* (Lehm.) P. Taylor, *Tridentaria* P.Taylor, *Pleiochasia* Kamieński, and *Lasiocaules* R.W.Jobson & Baleeiro (Jobson et al. 2017, 2018), and about 60 species.

From an evolutionary perspective, *Utricularia multifida* and *U. tenella* are interesting because they represent an early evolutionary *Utricularia* branch and have some unusual morphological characters, especially in their traps. For example, Lloyd (1932, 1942) found the traps of *U. multifida* and *U. tenella* to be “extremely curious” (see also Płachno et al. 2019a) because of trap structure. Reifenrath et al. (2006) proposed that the traps of *U. multifida* might not function with a low pressure-suction movement and might have permanent open trap doors, and therefore, their functioning differs from that in the rest of the *Utricularia* genus. Based on the trap architecture, these authors also suggested a close relationship between the genus *Genlisea* and the species *U. multifida*. Recently, Westermeier et al. (2017) showed no suction action, trapdoor movements or spontaneous firings of the *U. multifida* traps, so they proposed that the *U. multifida* trap type is passive. However, some “unusual” morphological characters of the *Polypompholyx*-*Tridentaria* lineage may not be plesiomorphic from an old lineage but could represent apomorphies as a result of a new specialisation. Taylor (1989) thought that the four-part calyx of the *Polypompholyx*-*Tridentaria* members may be an evolutionarily intermediate between *Genlisea* and other *Utricularia*; however, according to Jobson et al. (2018), it might be an independent apomorphy.

*Utricularia* species vary greatly in terms of their flower size, colour, and the occurrence of fragrance (Taylor 1989; see also Lowrie 2013 in the case of Australian *Utricularia*). In the subgenus *Polypompholyx*, there are evolutionary trends regarding the flower colour, morphology, and pollination syndrome. For example, Jobson et al. (2018), based on their parsimony-based ancestral state reconstruction (Jobson et al. 2017), examined the evolution of the colour of the lower lip of the corolla in the subgenus *Polypompholyx*, and, according to them, the earliest node shows a split between pink flowers in *Utricularia* sect. *Polypompholyx* + sect. *Tridentaria* and various shades of purple as being ancestral for sect. *Pleiochasia*. Later, other corolla colours (pink, apricot, white) appeared in this section (Jobson et al. 2017, 2018). Płachno et al. (2019b) proposed that the plesiomorphic *Utricularia* flower was bee pollinated, and that later other pollination syndromes evolved. Bird pollination (in the case of *U. menziesii*) is a recent apomorphy in the genus *Utricularia*, subgenus *Polypompholyx*. In addition, Reut and Jobson (2010) showed that the filiform corolla appendages in the *Utricularia* species from the subgenus *Polypompholyx* evolved independently three times—twice from the upper lip lobe and once from the lower lip lobe. These species are most probably sexually deceptive (pollination may occur by pseudocopulation) and their filiform corolla appendages have scent glands to attract pollinators (Płachno et al. 2016; Płachno et al. in preparation).

Unfortunately, the detailed floral micromorphology of species within *Utricularia* subgenus *Polypompholyx* has been poorly studied (Płachno et al. 2016, 2019b). The concentration and composition of the nectar sugars are known for only one species from the subgenus *Polypompholyx* (*U. menziesii*—Płachno et al. 2019b). Examining the floral micromorphology and nectar of additional species within the subgenus would provide us with further insight into the evolution of this early evolutionary lineage within the *Utricularia* genus. Such data will be useful for further studies on ancestral state reconstructions of pollination syndromes within the genus. Thus, our aims were (1) to determine whether the concentration and composition of nectar sugars in *U. multifida* and *U. tenella* are similar to those in other *Utricularia* species from the same subgenus (species that grow in the same environment *U. menziesii*—Płachno et al. 2019b; *U. violacea*—our data) and the subgenus *Utricularia* (data from Abrahamczyk et al. 2017), (2) to compare the nectary structure of *U. multifida* and *U. tenella* with that of other *Utricularia* species, and (3) because Lang (1901) described specially shaped floral trichomes in *U. multifida*, we sought to determine whether they can act as food trichomes (an alternative food reward for pollinators) for visiting insects or whether they perform other functions.

## Material and methods

### Plant material

Flowers of *Utricularia multifida* R.Br., *U. tenella* R.Br. (sect. *Polypompholyx*), and *U. violacea* R.Br. (sect. *Pleiochasia*) were collected from the Alison Baird Reserve (Yule Brook) in Western Australia by HL, FJN, and GRC. The flowers were fixed in a mixture of 2.5% (*v*/*v*) or 5% (*v*/*v*) glutaraldehyde with 2.5% (*v*/*v*) formaldehyde in a 0.05-M cacodylate buffer (pH 7.2; Sigma) or 70% (*v*/*v*) ethanol and sent to Poland for the morphological and histochemical studies (Jagiellonian University, Kraków). Additional plant material (*U. tenella* flowers in ethanol) was provided by the National Herbarium of Victoria, Melbourne, Australia.

### Floral structure and histochemical investigations

The distribution of the secretory glandular trichomes was determined by examining whole flowers (corollas) using a Nikon SZ100 stereoscopic microscope (Nikon Instruments Europe B.V., City, Country). The floral parts, namely the spurs, were examined using light microscopy and scanning electron microscopy. Fixed material was washed three times in a 0.1-M sodium cacodylate buffer and post-fixed in a 1% (*w*/*v*) osmium tetroxide solution at room temperature for 1.5 h. Dehydration was performed using a graded ethanol series, and infiltration and embedding using an epoxy embedding medium kit (Fluka). Semi-thin sections (0.9–1.0 μm) were prepared for light microscopy and stained for the general histology using aqueous methylene blue/azure II (MB/AII) for 1–2 min (Humphrey and Pittman 1974) and examined with an Olympus BX60 light microscope (Tokyo, Japan).

The hand sections were immersed in water and analysed using bright field and fluorescence microscopy. The material was tested for lipids, starch, and mucilage, using a saturated ethanol solution of Sudan III, an aqueous IKI (iodine-potassium iodide) solution, and a ruthenium red solution, respectively. The autofluorescence of the cuticle was observed under UV light, and the structure of the cuticle was studied on sections that had been stained with auramine O (Gahan 1984). For the scanning electron microscopy, the representative floral parts were fixed (as above), dehydrated, and subjected to critical drying point using liquid CO_2_. Then, they were sputter-coated with gold and examined at an accelerating voltage of 20 kV using a Hitachi S-4700 scanning electron microscope (Hitachi, Tokyo, Japan) at the Institute of Geological Sciences, Jagiellonian University in Kraków.

### Ultrastructure analysis

For the transmission electron microscopy (TEM), the flowers were fixed in a mixture of 2.5% (*v*/*v*) or 5% (*v*/*v*) glutaraldehyde with 2.5% (*v*/*v*) formaldehyde in a 0.05-M cacodylate buffer (pH 7.2; Sigma), washed three times in a 0.1-M sodium cacodylate buffer, and post-fixed in a 1% (*w*/*v*) osmium tetroxide solution at room temperature for 1.5 h. Dehydration using a graded ethanol series and infiltration and embedding using an epoxy embedding medium kit (Fluka) were followed. After polymerisation at 60 °C, sections for TEM were cut at 70 nm using a Leica ultracut UCT ultramicrotome, stained with uranyl acetate and lead citrate (Reynolds, 1963), and examined using a Hitachi H500 transmission electron microscope (Hitachi, Tokyo, Japan) at an accelerating voltage of 75 kV.

### Nectar collection and analysis

For each species (*U. multifida*, *U. tenella*, and *U. violacea*), the flowers from ten individual plants were collected in the field. Each flower was immediately stored on ice for transport to the laboratory. As it was not possible to extract free nectar using glass capillary tubes, washing flower spur in accordance with Morrant et al. (2009), who recommends flower wash for nectar collection from flowers with low nectar volumes in the field, was undertaken. The spur was removed from each flower with a scalpel blade under a magnifying glass. The spur was then placed in an Eppendorf tube and 100 μL Milli-Q water was added. Samples were vortex mixed before being centrifuged for 5 min; this was repeated three times. Later, the samples were analysed using high-performance liquid chromatography (HPLC) as in Płachno et al. (2019b).

## Results

### *Utricularia multifida* (Figs. 1, 2, 3, and 4)

**Fig. 1:**
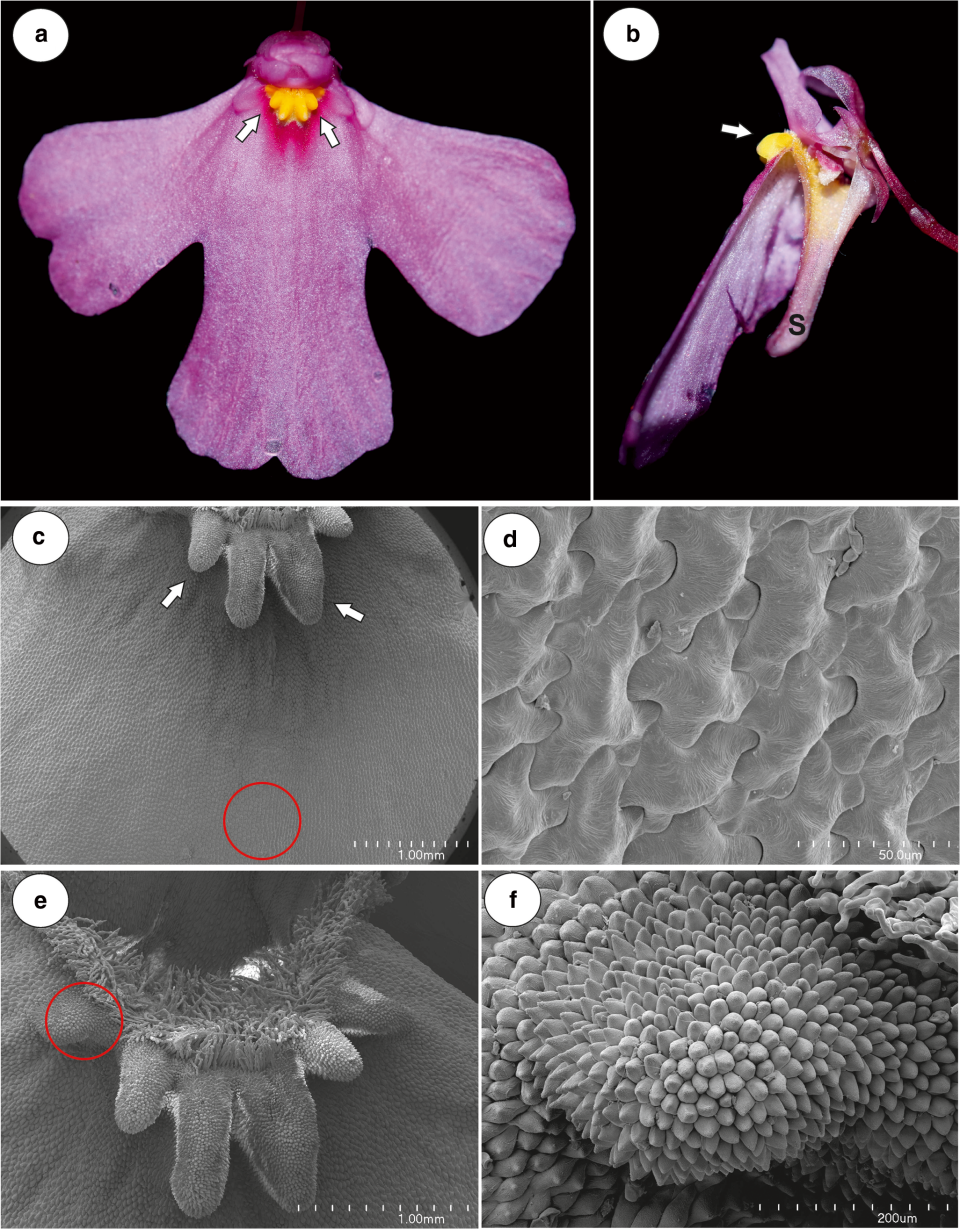
Floral morphology of *Utricularia multifida*. **a**–**b** General floral morphology; palate (arrow), nectar spur (s). **c** Micromorphology of the lower corolla lip; the palate (arrow), the encircled part shown at (**d**); bar = 1 mm. **d** Micromorphology of the epidermal cells of the lower lip; scale bar = 50 μm. **e** Morphology of the palate, note the numerous trichomes, which block the entrance to the throat, eclipsed part of palate is shown at higher magnification at 1F, scale bar = 1 mm. **f** Micromorphology of the epidermal cells of the palate, note the papillae and papillose cells; scale bar = 200 μm

**Fig. 2:**
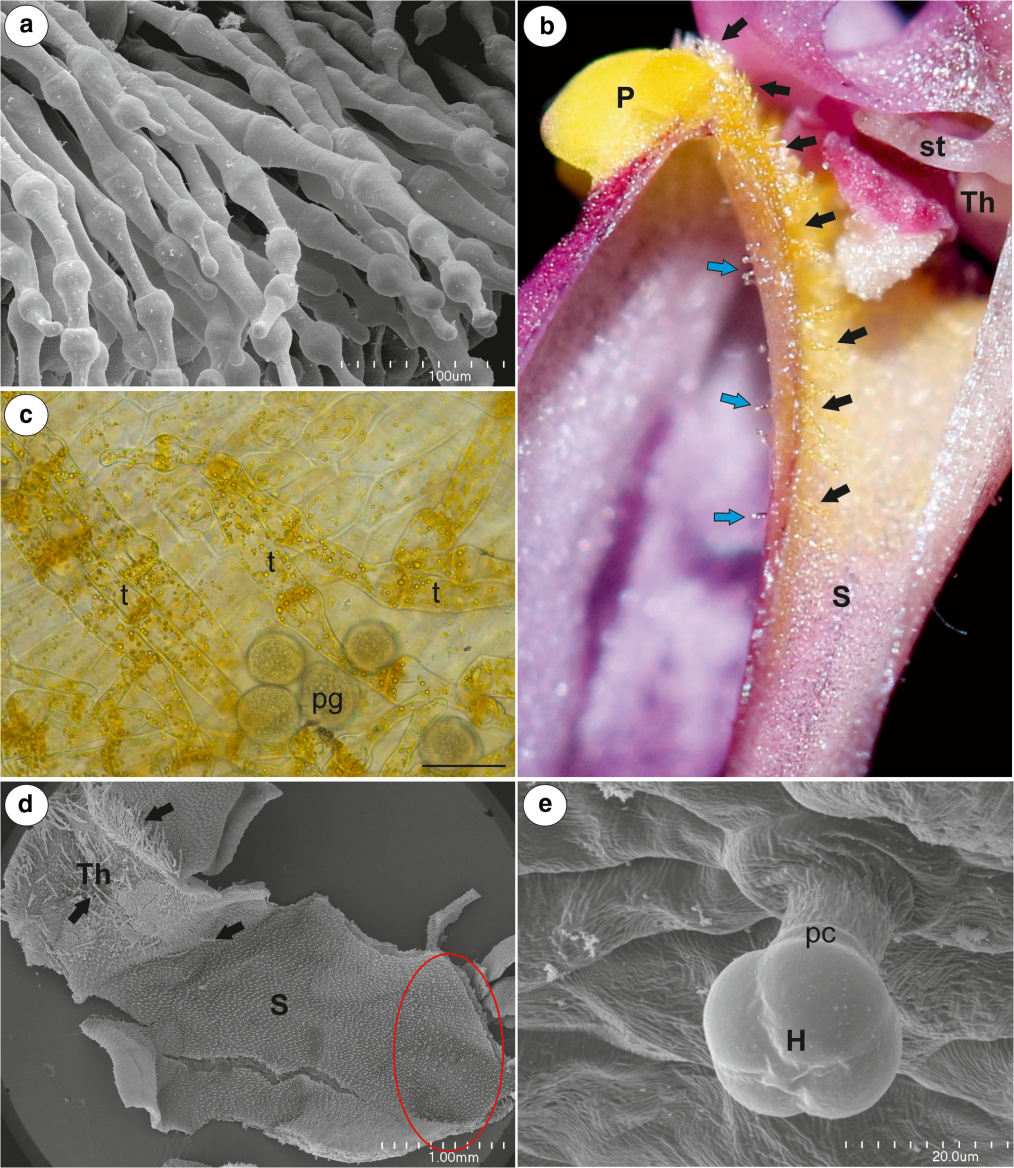
Floral morphology of *Utricularia multifida*. **a** Palate trichomes blocking the entrance to the throat; scale bar = 100 μm. **b** Section through the palate (P), throat (Th), and spur (S), internal trichomes (black arrows), external spur trichomes (blue arrows), stamen (st). **c** Accumulation of chromoplasts in the palate trichomes (t), pollen grain (pg); scale bar = 50 μm. **d** Morphology of the throat (Th) and spur (S), trichomes (arrows). Nectar trichomes in its apical part (ellipse); bar = 1 mm. **e** Micromorphology of an external spur trichome: head (H), pedestal cell (pc); scale bar = 20 μm

The corolla of *U. multifida* was pink with a yellow palate (Fig. 1a–b). It was bilabiate and spurred. The lower lip of the corolla was trilobate and flat and formed a wide landing platform for pollinators (Fig. 1a, c). Flat, “jigsaw-puzzle”-shaped cells covered the entire flat surface of the lower lip of the corolla (Fig. 1d). The palate had clearly visible parallel ridges; this part of the palate was covered by papillae with cuticular striations (on the ridges) or papillose cells with cuticular striations (Fig. 1e–f). The most prominent character of the palate was the occurrence of multi-celled trichomes (Figs. 1e and 2a–c), which had specific shapes (like an inflated balloon with constrictions, Fig. 2a). These trichomes blocked the entrance to the throat. The cells of these trichomes had many chromoplasts and contained some lipid droplets (Fig. 2c), which were also revealed by the Sudan staining. These trichomes did not have protein bodies or starch. These trichomes also occurred in the throat and in the basal part of the spur (Fig. 2b, d).

**Fig. 3:**
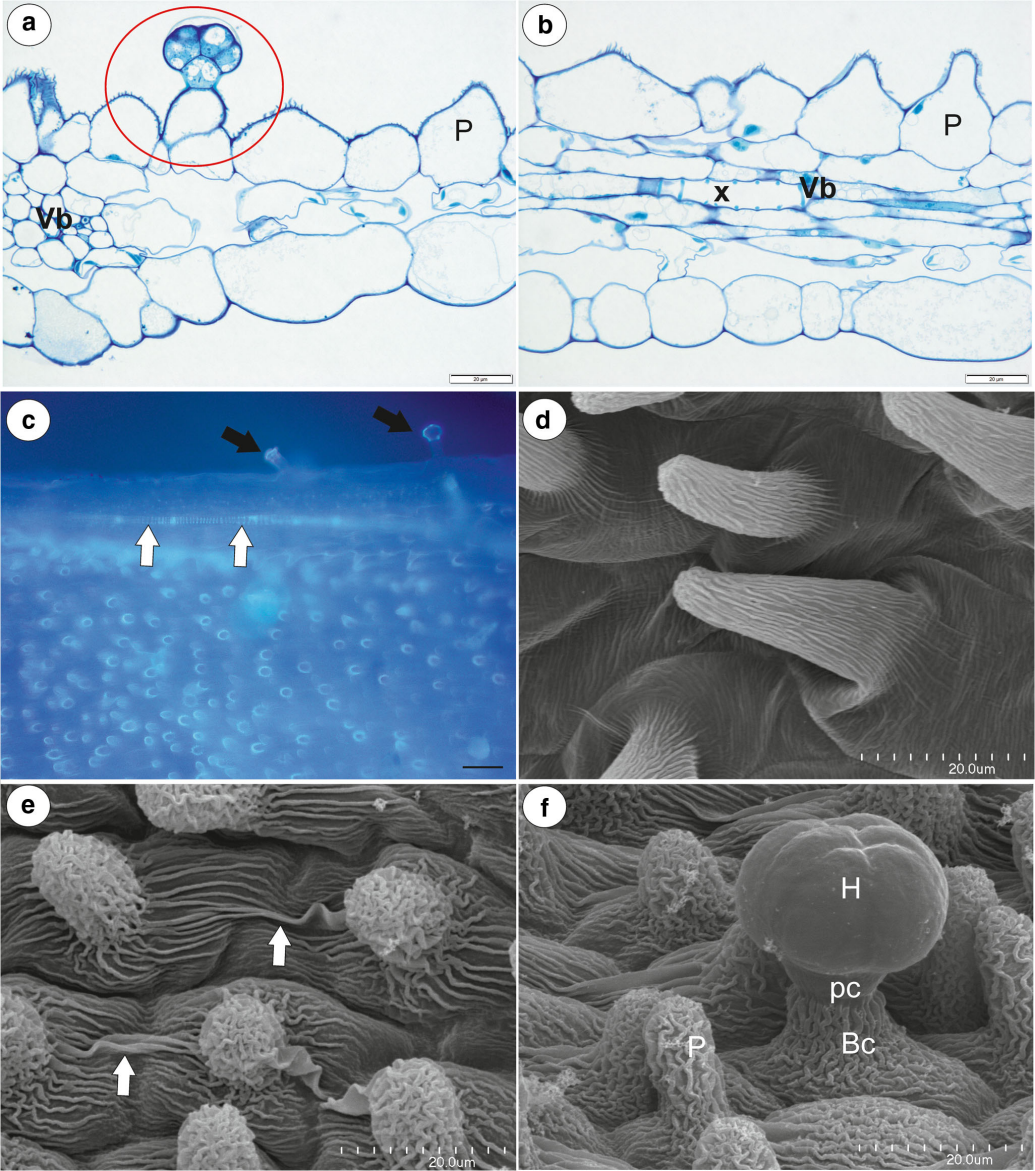
Anatomy and morphology of *Utricularia multifida*. **a**–**b** General anatomy of the spur, showing the vascular bundle (vb), tracheary xylem element (x), and nectary trichome (circle), papillae (P); scale bar = 20 μm. **c** Section of part of the spur, autofluorescence of the cuticle of the papilla; note external trichomes (black arrow), vascular bundles (white arrow); scale bar = 50 μm. **d** Micromorphology of the papillae from the basal part of the spur; scale bar = 20 μm. **e** Micromorphology of the papillae from the apical part of the spur, note that the cuticular striations of the neighbour cells are fused; scale bar = 20 μm. **f** Micromorphology of the nectary spur trichome: head (H), pedestal cell (pc), basal cell (Bc), papilla (P); scale bar = 20 μm

**Fig. 4:**
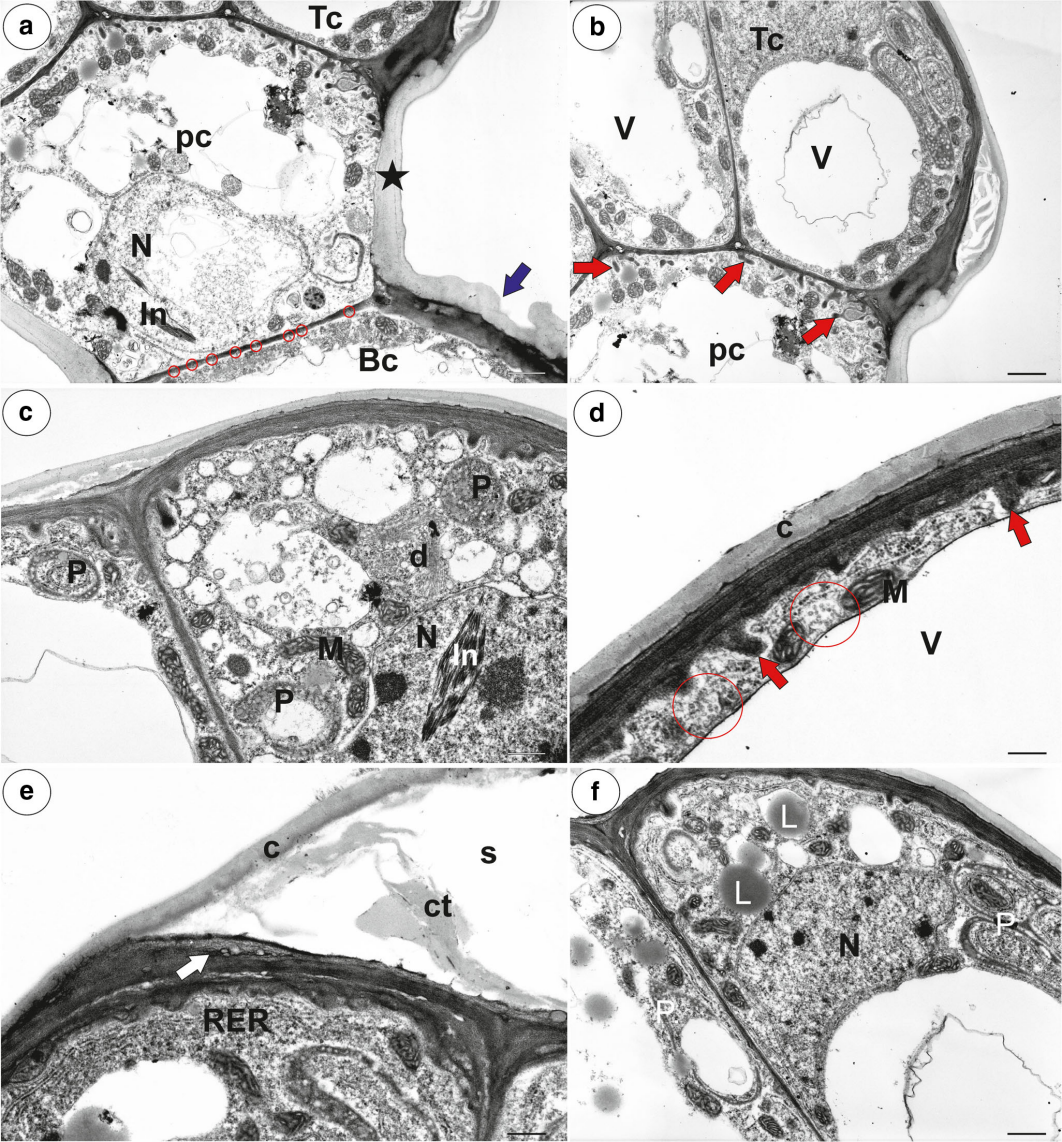
Ultrastructure of a nectary trichome from the spur of *Utricularia multifida*. **a**–**b** Ultrastructure of the basal cell (Bc), pedestal (pc), terminal cells (Tc); note the cell wall ingrowths (red arrows) in the pedestal cell: cuticular striations of a basal cell (blue arrow), plasmodesmata between a pedestal and a basal cell (circle), thickened impregnated anticlinal wall of a pedestal cell (star), nucleus (N), paracrystalline protein inclusion (In) in the nucleus of a terminal cell, vacuole (v); scale bar = 2 μm and scale bar = 2.2 μm. **c**–**f** Ultrastructure of the terminal cells; plastids (P), mitochondria (M), dictyosomes (d), cell wall ingrowths (red arrow), vacuole (v), lipid droplet (L), rough endoplasmic reticulum (RER), multivesicular bodies (circle), cuticle (c), material (ct) in the subcuticular space (s), the amorphous globules in the cutinised layer of the cell wall (white arrow); **c** scale bar = 1.2 μm, **d** scale bar = 0.65 μm, **e** scale bar = 1 μm, **f** scale bar = 1.5 μm

The cylindrical spur was directed downwards and parallel to the lower lip. Both the external and internal epidermis of the spur had small capitate glandular trichomes (Figs. 2b, d, e and 3a). The capitate glandular trichomes from the external spur surface consisted of a stalk cell, a pedestal cell and a four-celled head (Figs. 2e and 3c). In the transverse section, the wall of the spur was composed of several cell layers: the internal epidermis, layers of parenchyma cells and the outer epidermis (Fig. 3a–b). There were collateral vascular bundles in the ground parenchyma (Fig. 3a–c) and each contained both xylem and phloem elements. The internal epidermis formed papillae, which were unicellular with cuticular striations on their surface (Fig. 3a–f). The papillae from the basal part of the spur were slightly different from those of the apical part of the spur when the cuticular striations were developing. The papillae from the apical part of the spur had cuticular striations on the entire external cell surface (Fig. 3e–f) and the cuticular striations of the neighbouring cells were fused (Fig. 3e). There were no nectary stomata. Nectar trichomes occurred on the internal side of the spur on its apical part (Figs. 2d and 3a, f). Each nectar spur trichome was composed of a single basal cell that formed a unicellular stalk, a pedestal cell (barrier cell), and a multi-celled head (Fig. 3f). The basal cell had prominent cuticular striations on its surface (Figs. 3f and 4a). There were numerous plasmodesmata in the transverse walls between the stalk cell and the pedestal cell (Fig. 4a). The pedestal cell had a thick radial wall, which was impregnated with cutin (Fig. 4a). The cytoplasm of the pedestal cell contained a nucleus and the usual organelles (mitochondria, plastids, and profiles of the endoplasmic reticulum; Fig. 4a). In addition, there were also lipid droplets. The reticulate cell wall ingrowths occurred on the transverse wall and partially on the lateral wall of the pedestal cell (Fig. 4b). The terminal (head) cells had large vacuoles (Fig. 4b–d). Mitochondria and plastids were numerous (Fig. 4c–f). The plastids were cup-shaped and contained numerous small lipid globules (Fig. 4c and f). These organelles were associated with the rough endoplasmic reticulum. Only few dictyosomes were observable (Fig. 4c). Lipid droplets were visible in the cytoplasm (Fig. 4f). The small multivesicular bodies in the thin layer of cytoplasm between the plasmalemma and vacuole were observed (Fig. 4d). There were small cell wall ingrowths only on the inner surface of the outer wall (Fig. 4d). The cuticle of terminal cells was very thick. Amorphous globules occurred in the cutinised layer of the cell wall (Fig. 4e). The cuticle frequently became distended and separated from the cell walls and formed a subcuticular space (Fig. 4e). We observed some lipid or cutin material between the cuticle and the cell wall (Fig. 4e). Hexose-dominated nectar was detected in the flower spurs (fructose 54 ± 0.5%, glucose 46 ± 0.5%; sugar concentration, fructose 42.2 ± 5.5 μg flower^−1^, glucose 36.2 ± 5.0 μg flower^−1^). The flowers of *U. multifida* were visited by European honeybees (observation done by Dr Marion Cambridge, during the flowering period between September and November 2018 in Alison Baird Reserve).

### *Utricularia tenella* (Fig. 5)

**Fig. 5:**
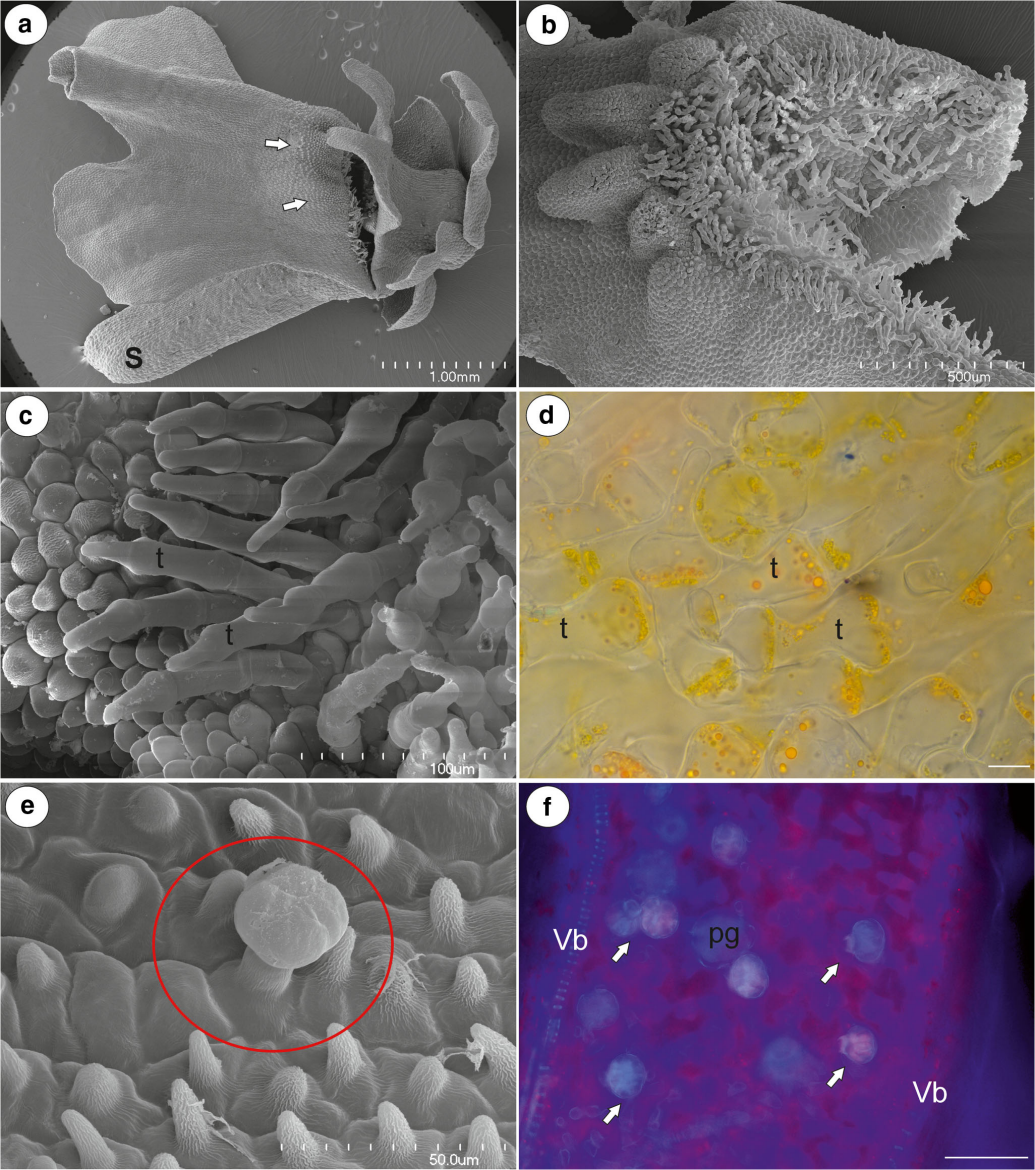
Floral morphology and anatomy of *Utricularia tenella*. **a** General floral morphology; palate (arrow), nectar spur (s); bar = 1 mm. **b** Morphology of the palate; note the numerous trichomes blocking the entrance to the throat; bar = 500 μm. **c** Palate trichomes blocking the entrance to the throat; scale bar = 100 μm. **d** Accumulation of chromoplasts in the palate trichomes (t); scale bar = 10 μm. **e** Micromorphology of the internal spur surface; note the numerous papillae and nectary trichome (circle); scale bar = 50 μm. **f** Section of part of the spur, autofluorescence of the cuticle of the nectar trichomes (arrow), vascular bundles (vb), pollen grain (pg); scale bar = 50 μm

The flower anatomy (spur anatomy) and micromorphology (epidermal cell types, papillae, trichome structure, and distribution, Fig. 5a–f) of *U. tenella* were very similar to that of *U. multifida*. The flower of *U. tenella* (Fig. 5a) resembled a smaller version of *U. multifida*. The palate trichomes (Fig. 5b–c) contained many chromoplasts and some lipid droplets (Fig. 5d). There were multicellular, capitate, shortly stalked, glandular trichomes, and papillae within the spur (Fig. 5e–f). The cuticular striations of the trichome stalk cell were less developed (Fig. 5e) compared with those of *U. multifida* (Fig. 3f). Hexose-dominated nectar was detected in the flower spurs (fructose 54 ± 1.2%, glucose 46 ± 1.2%; sugar concentration, fructose 8.1 ± 3.0 μg flower^−1^, glucose 6.7 ± 2.4 μg flower^−1^).

### *Utricularia violacea* (Fig. 6)

**Fig. 6:**
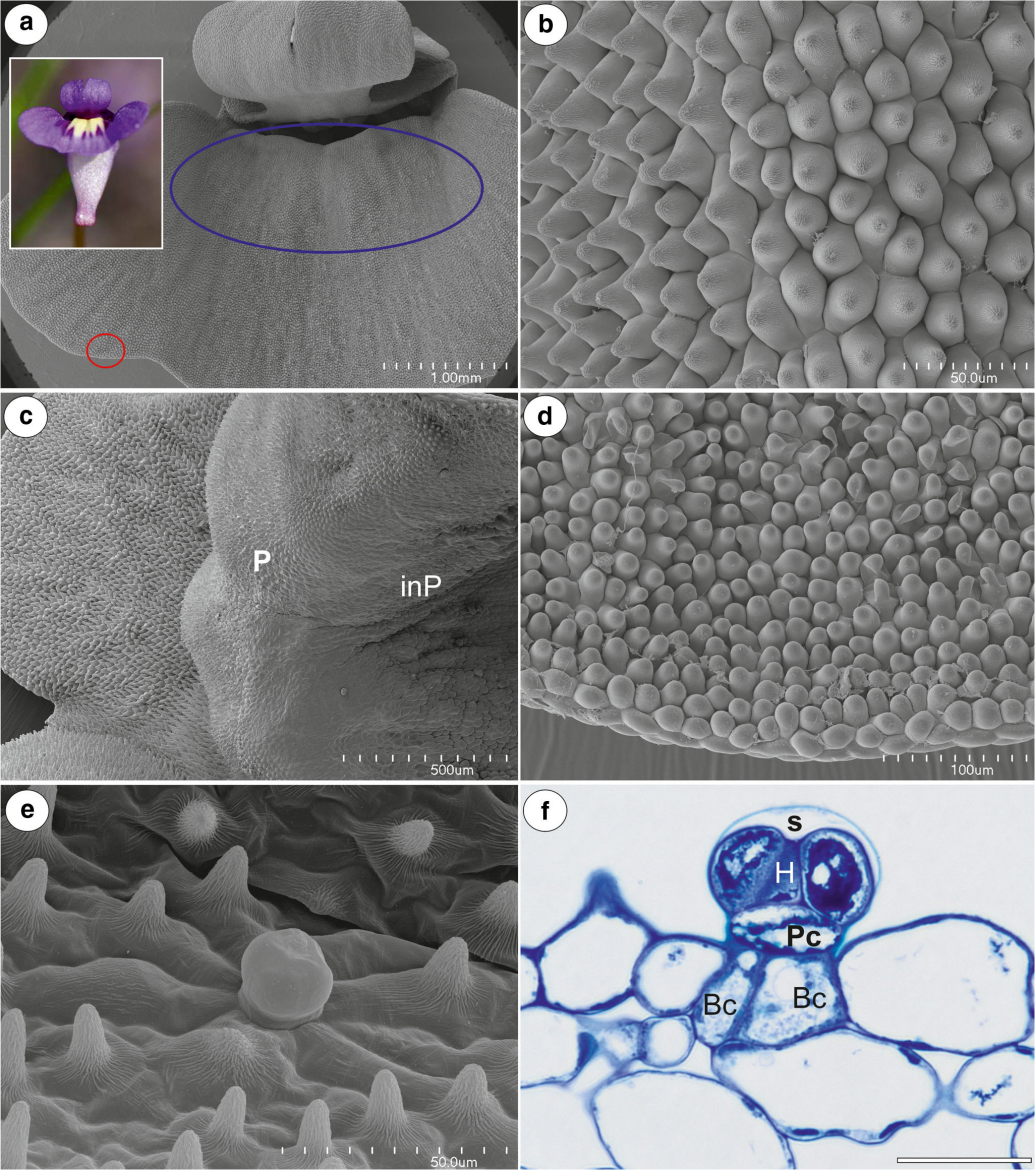
Floral morphology and anatomy of *Utricularia violacea*. **a** General floral morphology; palate (blue eclipsed), margin of the lower lip (red eclipsed part, is shown at higher magnification at 6D); bar = 1 mm. **b** Micromorphology of the palate surface; note the papillae with cuticular striations; scale bar = 50 μm. **c** Micromorphology of the palate, note the external part of palate with papillae (P), and glabrous internal part of palate (InP); scale bar = 500 μm. **d** Micromorphology of the epidermal cells of the lower lip; scale bar = 100 μm. **e** Micromorphology of the internal spur surface; note the numerous papillae and nectary trichome; scale bar = 50 μm. **f** General structure of the nectar trichome; note that the head cells of the trichomes stain intensely with MB/AII–terminal = head cells (H), pedestal cell (Pc) and two basal cells (Bc); scale bar = 10 μm

The corolla of *U. violacea* was blue-violet with a yellow palate with dark violet marks (Fig. 6a framed part). It was bilabiate and spurred. The lower lip of the corolla formed a wide landing platform for pollinators (Fig. 6a). The palate had clearly visible protrusions; this part of the palate was covered by papillae with cuticular striations (Fig. 6b). The inner part of the palate was glabrous (Fig. 6c). Papillose cells covered the entire flat surface of the lower lip of the corolla (Fig. 6d). Transverse sections showed that the wall of the spur was composed of several cell layers: the internal epidermis, layers of parenchyma cells, and the outer epidermis. Within the spur were multicellular, capitate, sessile-glandular trichomes, and papillae (Fig. 6e–f). Each nectar spur trichome was composed of two basal cells, a pedestal cell (barrier cell), and a multi-celled head (Fig. 6f). The terminal (head) cells were transfer cells; there were cell wall ingrowths on the inner surface of the outer wall and on the inner walls between the terminal cells (not shown). The thick cuticle frequently became distended and separated from the cell walls of the head cells to form a subcuticular space (Fig. 6f). Hexose-dominated nectar was detected in the flower spurs (fructose 58 ± 0.3%, glucose 42 ± 0.3%; sugar concentration, fructose 20.5 ± 1.5 μg flower^−1^, glucose 14.7 ± 1.2 μg flower^−1^).

## Discussion

We show that all three examined species, *Utricularia multifida*, *U. tenella*, and *U. violacea*, had hexose-dominated (fructose + glucose) nectar, and this suggests that they are pollinated by similar pollinators. However, Abrahamczyk et al. (2017) have shown that low nectar sucrose proportion (i.e. hexose-dominated nectar) may indicate generalist-pollinated plants. The *U. multifida* flowers were visited by European honeybees, which are probably also pollinators of the other species. Carow (Fig. 22.2a in Cross et al. 2018) also observed a halictid bee visiting a *U. multifida* flower. To date, the nectar of only one other species from the subgenus *Polypompholyx* has been examined—*U. menziesii* (Płachno et al. 2019b), which is probably bird-pollinated (its flowers are visited by a bird—the Western spinebill, *Acanthorhynchus superciliosus*, Lambers et al. 2014; Lowrie 2013). *Utricularia menziesii* also has hexose-dominated nectar; the occurrence of this kind of nectar does not exclude insects as additional visitors of the flowers of *U. menziesii*, but such an observation is lacking. Unfortunately, other data about the sugar composition in *Utricularia* nectar are limited to only three South American species: *U. alpina* Jacq*.*, *U. reniformis* A.St.Hil., and *U. nephrophylla* Benj. (Abrahamczyk et al. 2017), which are classified as pollinated by bees and wasps. These species belong to sect. *Orchidioides*, subgenus *Utricularia* (Rodrigues et al. 2017; Silva et al. 2018). According to Abrahamczyk et al. (2017), although both *U. nephrophylla* and *U. reniformis* have hexose-dominated nectar, only sucrose was detected in the nectar of *U. alpina*. Among these species, pollinators are only known for *U. reniformis* – *Xylocopa* sp. and *Bombus* sp. (Clivati et al. 2014). Hobbhahn et al. (2006) examined nectar production in *U. purpurascens* and *U. reticulata* (both from section *Oligocista*, subgenus *Bivalvaria*). Nectar was detected in *Genlisea* spurs (Fleischmann 2012), and it is produced by small capitate trichomes in *Genlisea violacea* spurs (Aranguren et al. 2018). *Genlisea violacea* nectar is mainly composed of fructose and glucose, which is similar to assessed *Utricularia* species, and its quantities are stable during the day (Aranguren 2016). Abrahamczyk et al. (2017) provided data about the nectar in five species of *Pinguicula*. Species that are classified as pollinated by butterflies, *Pinguicula macrophylla* Kunth, and *Pinguicula moctezumae* Zamudio & R.Z. Ortega, have fructose-dominated nectar, whereas species that are classified as pollinated by bees and wasps have sucrose-dominated nectar (*Pinguicula gigantea* Luhrs) or hexose-dominated nectar (*Pinguicula leptoceras* Rchb.). *Pinguicula alpina* L., which is pollinated by flies, have sucrose-dominated nectar.

It should be added that Abrahamczyk et al. (2017) presented evidence that nectar sucrose proportion is an adaptation in nectar evolution to pollinator group in asterids, but these authors also suggested that adaptation to pollinators is not a sufficient explanation on its own. We showed that species from *Utricularia* early evolutionary lineage (subgenus *Polypompholyx*) had hexose-dominated nectar, but occurrence of sucrose-rich nectar in species from advanced evolutionary lineage (*U. alpina*, sect. *Orchidioides*, subgenus *Utricularia*; Abrahamczyk et al. 2017) may indicate that sugar composition is not “phylogenetically constrained” (phylogenetic conservatism in sugar composition) in the case of *Utricularia*.

According to Lang (1901), the edge of the *U. multifida* throat is surrounded by a wreath of peculiarly shaped trichomes. He described this as “hairs are very rich in plasma and the outer walls of their cells are only weakly cutinized; the transverse walls between the individual cells are extremely delicate; they are not cutinized.” We found that this type of trichomes also occurs in *U. tenella*. It is possible that they block the entrance into the throat and nectariferous spur to visiting insects that do not fit their pollination syndrome (illegitimate visitors); this may protect them from having their nectar stolen. Because these trichomes were yellow and rich in chromoplasts, we suggest that they form an additional visual attractant for bees. Because we wanted to determine whether they could be food trichomes (an alternative food reward for pollinators), we checked for the occurrence of starch, protein bodies, and lipid droplets. Because the cells of these trichomes did not accumulate starch and protein bodies and contained only some lipid droplets, we have no evidence that they play the role of food trichomes and suggest that they are a visual and tactile guide for their pollinators. The occurrence of lipid droplets is relatively rare in edible trichomes and has only been recorded in some species of Orchidaceae, e.g. in *Cyanaeorchis arundinae* (Pansarin and Maciel 2017) and *Maxillaria* (Davies et al. 2000). Lang (1901) was thought that these trichomes were undoubtedly used for insect pollination; however, he also proposed that they might prevent the penetration of rain. Jachuła et al. (2018) proposed that the non-glandular trichomes of the *Linaria vulgaris* palate are involved in protecting against airborne fungal propagules or dust particles; therefore, they may also play a similar role here. However, *U. violacea*, which grows together with *U. multifida* and *U. tenella*, does not have these trichomes, whereas other species from the subgenus *Polypompholyx* (*U. menziesii*, *U. uniflora*, and *U. dichotoma*) exhibit many unicellular trichomes at the palate and throat (Płachno et al. 2016, 2019b). The histochemical tests indicated that these trichomes did not produce mucilage or proteins, but that they did have chromoplasts. Płachno et al. (2019b) proposed that these trichomes are a tactile signal. In *Genlisea violacea*, there are non-glandular trichomes on the palate or throat (Aranguren et al. 2018). However, these trichomes are different from the trichomes of *U. multifida* and *U. tenella* which have thick cuticular striations (well visible under UV light in *Genlisea*, see Fig. 4 in Aranguren et al. 2018) and a different cell shape. In *Pinguicula* flowers, there are numerous non-glandular multicellular trichomes at the palate and in the throat. These trichomes are useful taxonomic characters and aid in species identification (Casper 1966). According to Fleischmann (2016), these trichomes are considered “feeding hairs,” and in some *Pinguicula* species, these trichomes (a cluster of yellow trichomes on the palate) mimic a stamen or pollen. Examined species did not have glandular trichomes at the palates, in contrast to some species of *Utricularia* (Płachno et al. 2017b) and *Genlisea* (Płachno et al. 2018a).

The floral nectaries in the analysed species are not much different from those of previously investigated *Utricularia* species in their anatomy and micromorphology (Clivati et al. 2014; Płachno et al. 2016, 2017a, 2018b, 2019b). The spur is the organ in which nectar is produced and stored. The nectar is produced by small capitate trichomes, which have a similar architecture across the genus (Płachno et al. 2018b). The only difference is the presence of two basal cells in the trichomes of *U. violacea*. Glandular capitate trichomes with two basal cells were recorded in *Byblis* (Lloyd, 1942), which is a genus that was previously considered to be related to Lentibulariaceae, but which was reclassified into Lamiales (APG IV 2016; Schäferhoff et al. 2010). The ultrastructure of the nectary trichomes in *Utricularia multifida* is very similar to that of species from section *Utricularia* (Płachno et al. 2018b); this is evidence for a conservative construction of the nectary cells in this genus. The cup-shaped plastids that have been recorded in the glandular trichome cells of *U. multifida* are typical for the secretory cells of nectary trichomes (Płachno et al. 2018b) and palate trichomes in *Utricularia* (Płachno et al. 2017b). The presence of lipid droplets in the cytoplasm in the nectary trichome cells of *U. multifida* suggests that it may enrich the nectar with lipids. This was suggested for the nectaries of various plant species from other families (Machado et al. 2017). The low densities of dictyosomes that were observed in the glandular trichome cells of *U. multifida* indicate that nectar secretion occurs via an eccrine mode. The presence of cell wall ingrowths and numerous mitochondria (Gunning and Pate 1969) in the glandular trichome cells also supports this. This mode of secretion was previously proposed by Płachno et al. (2018b) for the nectary trichomes of *Utricularia* species from section *Utricularia*. Vassilyev (2010) proposed that sugars cross the plasma membrane via an active transport, which characterises an eccrine secretion. Other authors also favour an eccrine mode of the secretion of nectar, e.g. Lüttge and Schnepf (1976), Nepi (2007), and Paiva (2012). Based on evidence available from phylogenetic studies of this genus (e.g. Jobson and Albert 2002; Jobson et al. 2017, 2018; Müller and Borsch 2005; Silva et al. 2018) and recent studies on their floral and nectary structure (e.g. Clivati et al. 2014; Lowrie 2013; Płachno et al. 2016, 2017a, 2018b, 2019b; Taylor 1989, and our data), we suggest that an early ancestor of *Utricularia* had a nectariferous spur flower with a lower lip that formed a wide landing platform for its bee pollinators.

## Conclusions

*Utricularia multifida*, *U. tenella*, and *U. violacea* exhibit traits indicative of the bee pollination syndrome (melittophily): closed, zygomorphic flowers with vivid colours, and hexose-dominated (fructose + glucose) nectar inside the spur of the corolla. However, the occurrence of hexose-dominated nectar may also indicate a broader spectrum of pollinators. Both *U. multifida* and *U. tenella* have trichomes that block the entrance into the throat and the nectariferous spur to visiting insects that do not fit their pollination syndrome. Because these trichomes are rich in chromoplasts and have a specific shape, we suggest that they are additional visual and tactile attractants for pollinators.
